# Effect of Zr Microalloying on the Microstructures and Strengthening Mechanism of As-Cast Al-Fe-Zr Alloys

**DOI:** 10.3390/ma13214744

**Published:** 2020-10-23

**Authors:** Jieyun Ye, Renguo Guan, Hongjin Zhao, Changwei He, Kezhi Xiong

**Affiliations:** 1 Faculty of Material Metallurgy and Chemistry, Jiangxi University of Science and Technology, Ganzhou 341000, China; 9120130116@jxust.edu.cn (J.Y.); 9119960101@jxust.edu.cn (H.Z.); 1320181650@mail.jxust.edu.cn (C.H.); 1320181654@mail.jxust.edu.cn (K.X.); 2 Engineering Research Center of Continuous Extrusion, Dalian Jiaotong University, Ministry of Education, Dalian 116028, China

**Keywords:** Al-Fe-Zr alloys, microalloying, microstructure, mechanical properties

## Abstract

The microstructure and mechanical properties of Al-0.35Fe alloys with a series of different zirconium (Zr) additions from 0.1 to 0.4% are investigated by optical microscopy, scanning electron microscopy, transmission electron microscopy and tensile testing. The as-cast structure of the alloys varies with the Zr content. When the content of Zr is 0.1%, Zr dissolves into the aluminum (Al) matrix completely and iron (Fe) concentrates along the boundary in a network of eutectic Al_3_Fe. With the increase in Zr content to 0.2% and above, nanoscale Al_3_Zr particles appear in the alloy. With the Zr content increasing from 0.1 to 0.4%, the grain size of the Al matrix decreases from 73 to 23 μm. The morphology of the eutectic Al_3_Fe phase changes from short rod-like to an agglomerated structure consisting of finer and shorter rod-like shapes. The tensile and yield strengths increase while the total elongation decreases with increasing Zr content. The strengthening mechanism of the alloy can be attributed to the combination of fine-grain, solution and second-phase strengthening.

## 1. Introduction

Fe is considered to be one of the most harmful impurities in Al alloys. Because of its low solubility in Al alloys, Fe often precipitates in the form of a needle-like β-Fe phase during solidification [1,2,3,4,5]. The β-Fe phase distributes at grain boundaries and can easily cause stress concentration. Eventually, it leads to a decrease in strength and toughness [6,7]. However, the removing process for Fe impurities is difficult and expensive. Considering the good heat resistance and high hardness of the Fe-rich phase, more and more research has become focused on the modification of the Fe-rich phase in recent years [8,9,10]. Wang et al. [11] refined Al_3_Fe to the nanoscale through shear deformation by means of rheo-extrusion, thereby improving the mechanical properties of an Al-1Fe alloy. Stolyrov et al. [12] significantly refined Al_3_Fe by equal-channel angular pressing and the tensile strength and elongation increased as a result. Li et al. [13] found that with an increase in the magnetic field, the nuclei number of the primary Al_3_Fe increased, resulting in a decrease in the Al_3_Fe phase. The addition of alloy elements has also been studied to improve the Fe-rich phase.

Rahul et al. [14] found that the addition of cobalt to Al-Fe alloys could hinder the diffusion behaviors of Fe and inhibit the growth of the Al_3_Fe phase. Zhang et al. [15] demonstrated that the addition of scandium significantly refined Al_3_Fe to the nanoscale. Shi et al. [8] indicated that adding 0.3% (all compositions quoted in this work are in wt.% unless specified otherwise) of rare-earth elements into an as-cast Al-1Fe alloy could improve the morphology of the Fe-rich phase and transform it from needle flakes to short rod shapes. Zr is a perfect addition to Al alloys, since it can form a dispersive and fine precipitated phase which is coherent with the matrix. Combined microalloying is often designed in Al alloys through Sc, Er, Zr addition. Wang et al. [16] and Deng et al. [17] suggested that the formation of Al_3_ (Sc, Zr) prevents the unsuitable diffusion and promotes the heterogeneous nucleation for α-Al during solidification. The recrystallization behavior of Al-3Cu (wt.%) at 500 °C was fully inhibited with co-additions of Er and Zr and leading to a significant grain refinement [18]. Besides, the Zr tends to segregate to θ’ interfaces and grain boundaries, which improves θ’ stability and induces precipitation of particles (Al_3_ (Sc, Zr)) at grain boundary [19,20]. Hence, the strength, creep resistance and low-stress corrosion can be improved by Zr microalloying. As for Al-Fe alloy after hot-extrusion, the addition of Zr also inhibits recrystallization and improves the heat resistance [21]. However, the effect of Zr microalloying on Al-Fe alloys during solidification has not been studied.

At present, for a large amount of aluminum alloy resource recovery, the recovery cost increases due to the high Fe content. How to make full use of Al-Fe alloy is a difficult problem and challenge [22,23]. So this research focuses mainly on the effect of Zr microalloying on the improvement of Fe phase morphology, mechanical properties and strengthening mechanism. Al-Fe-Zr alloys with a series of different Zr additions from 0.1 to 0.4% are prepared using a copper mold. The microstructure is investigated and the room temperature compressive properties are assessed. Furthermore, the work is extended to understanding the grain refining mechanism, the modification mechanism of the Fe-rich phase and the strengthening mechanism of Zr microalloying Al-0.35Fe alloys.

## 2. Materials and Methods

Al-0.35Fe alloys with a series of Zr additions from 0.1 to 0.4% were prepared using commercial pure aluminum (99.99%), with master alloys of Al-10Zr and Al-10Fe (mass fraction, %). For each composition, pure Al was melted in a crucible resistance furnace under the protection of argon gas. Subsequently, various amounts of master alloys were added into the melt and maintained for several minutes. A preheated ceramic rod was utilized to stir the melt in order to homogenize Fe and Zr. After degassing and deslagging, the molten alloy was poured into a preheated metal mold with a size of Φ40 × 130 mm. The alloy composition was measured by an Iris Advantage 1000 inductively-coupled plasma spectrometer (Thermo Jarrel Ash Corporation, Franklin, MA, USA), as shown in Table 1 [21].

The samples for microstructural observation were prepared in specimens with dimensions of Φ10 × 10 mm, extracted from the central part of the casting ingots. The sample surfaces were polished and etched by a Keller alcohol solution for observation. The microstructures were examined using a metallurgical DSX500 microscope (OLYMPUS, Tokyo, Japan), and the grain size distribution was statistically measured by analysis software (IMAGE-PRO-PLUS 6.0) according to the ASTM E112-2013 standard. The morphology and distribution of the phase were investigated through the combination of an Ultra Plus scanning electron microscope (Carl Zeiss AG, Oberkochen, Germany) equipped with X-ray energy dispersive spectrometers. A FEI G2 20 transmission electron microscope (FEI Company, Hillsboro, OR, USA) was used to investigate the precipitates, grain boundaries and other substructures. Thin foils for the transmission electron microscopy analysis were prepared by electrospark wire electrode cutting sample pieces from the extruded materials and polishing them mechanically to ~50 µm. Discs with a 3 mm diameter were punched and dimpled to 60 µm, followed by twin-jet electropolishing (Struers ApS, Ballerup, Denmark) at 20 V. A solution of one third (volume fraction) nitric acid and two-thirds methanol at −25 °C was applied during electropolishing.

Tensile testing of the alloy ingots was carried out using a CMT5105 electronic tensile testing machine (MTS Test Technology Co. Ltd, Jinan, China) at a tensile velocity of 2 mm/min. Round tensile specimens were prepared according to the GB/T 228.1-2010 standard and with the gage length of 25 mm, which were cut out by electric discharge machining. An electronic extensometer was used to record a load–deformation curve within the gauge distance automatically and then the yield strength was calculated on the basis of the load–deformation curve. Tensile properties were tested at room temperature, and to ensure the accuracy of the test data, tensile samples for each Zr additions were tested 3 times.

## 3. Results

### 3.1. Effect of Zr Content on As-Cast Microstructures and Phase Composition

**Figure 1:**
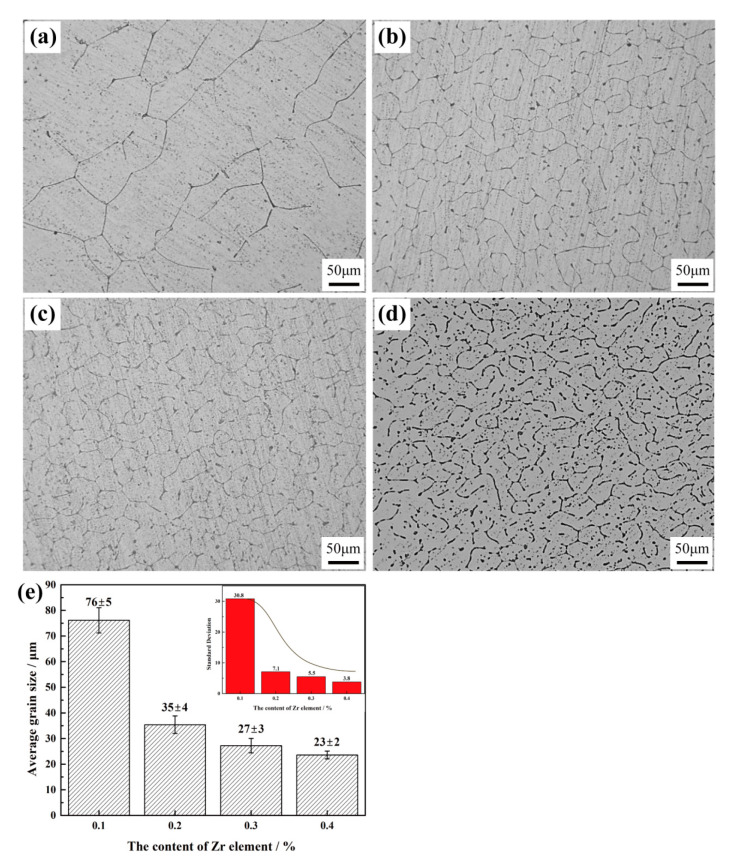
Optical microscopy images of as-cast alloys: (**a**) Al–0.35Fe–0.1Zr; (**b**) Al–0.35Fe–0.2Zr; (**c**) Al–0.35Fe–0.3Zr; (**d**) Al–0.35Fe–0.4Zr; (**e**) the average grain size and standard deviation of alloys.

Figure 1 shows the optical microstructure map of the as-cast Al-Fe-Zr alloy, etched using Keller’s reagent. The as-cast microstructure of Al–0.35Fe–0.1Zr alloy had an irregular bulky shape and formed a rough dendritic structure. The average size of the α-Al grains was 76 ± 5 μm. However, the addition of 0.2% Zr resulted in better modification effects in Al–0.35Fe–0.2Zr alloy (Figure 1b) and the average size of the α-Al grains decreased to 35 ± 4 μm. When the content of Zr increased to 0.3 and 0.4%, the average grain sizes were 27 ± 3 μm and 23 ± 2 μm, respectively (Figure 1c,d). Figure 1e shows the average grain size of the as-cast microstructure. With the increase in Zr content, the average grain size of the alloy decreases gradually. From the standard deviation results (Figure 1e), the grain size becomes more uniform when the Zr content increases to 0.3% and 0.4%, and the growth morphology of α-Al changed from dendritic to fine equiaxial crystals. In conclusion, the as-cast microstructures were efficiently refined by the addition of Zr from 0.1 to 0.4%. The cast morphology was optimized significantly. It is worth mentioning that when the Zr content was increased from 0.1 to 0.2%, the refining effect was obvious. However, the refining effect change was not obvious when the Zr content was further increased.

**Figure 2:**
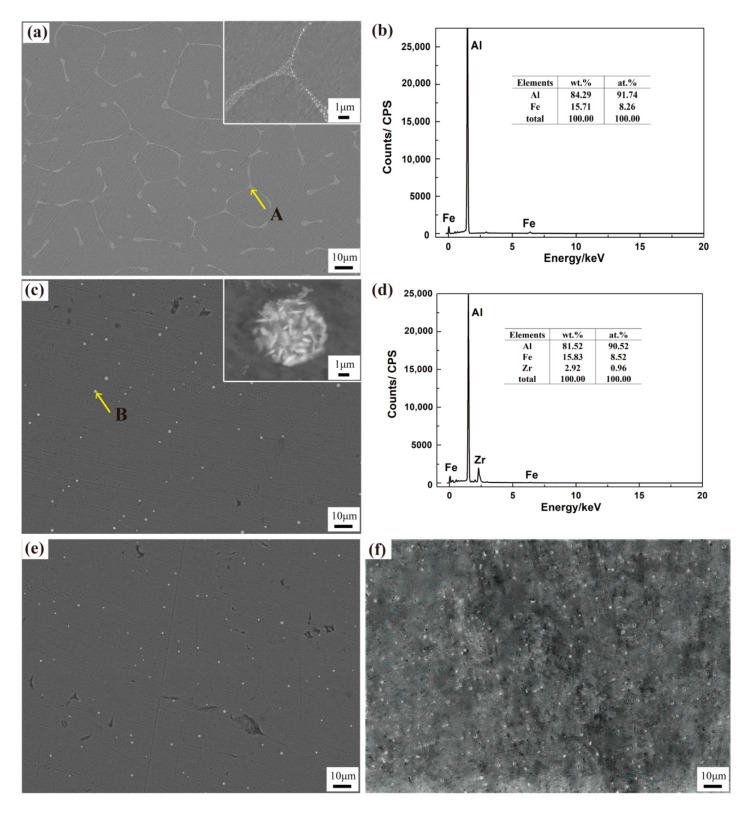
Scanning electron microscope images and energy-dispersive X-ray spectroscopy (EDS) of as-cast alloys: (**a**) Al–0.35Fe–0.1Zr alloy; (**b**) EDS of the A points; (**c**) Al–0.35Fe–0.2Zr alloy; (**d**) EDS of the B points; (**e**) Al–0.35Fe–0.3Zr alloy; (**f**) Al–0.35Fe–0.4Zr alloy.

The scanning electron microscopy and energy-dispersive X-ray spectroscopy (EDS) results are shown in Figure 2. Al–0.35Fe–0.1Zr alloy is composed of α-Al matrix and a brighter-hued white second phase distributed at the grain boundaries. The average length of the second phase (red arrow) is 300 nm. The EDS analysis shows that the second phase is composed of Fe and Al (Figure 2e). In Al–0.35Fe–0.2Zr alloy, the elements of the second phase distributed at the grain boundaries are Al, Fe and Zr. The morphology of the second phase changes to granulate and it shows discontinuity. After magnification, it is found that the particles are actually spherical clusters composed of a large number of small and short rod-shaped phases. The average size of the spherical clusters is 1.2 μm and the short rod-shaped phases are 190 nm. When Zr content increased to 0.3 and 0.4%, spherical clusters become more evenly distributed.

**Figure 3:**
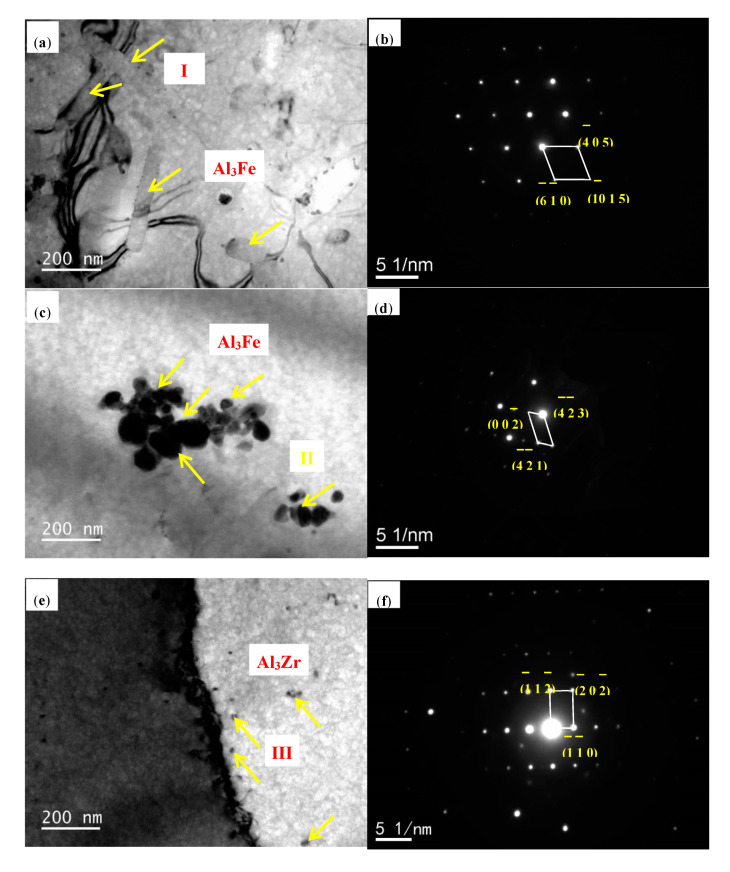
Transmission electron microscopy images and of as-cast alloys: (**a**) Al–0.35Fe–0.1Zr alloy; (**b**) EDS analysis of point I, showing the diffraction pattern of Al_3_Fe phase; (**c**) Al–0.35Fe–0.2Zr alloy; (**d**) EDS analysis of point II, showing the diffraction pattern of Al_3_Fe phase; (**e**) Al–0.35Fe–0.2Zr alloy; (**f**) EDS analysis of point III, showing the diffraction pattern of Al_3_Zr phase.

Figure 3 shows the TEM micrograph of Al–0.35Fe–0.1Zr alloy and Al–0.35Fe–0.2Zr alloy. It can be seen that the rod-shaped phase in Al–0.35Fe–0.1Zr alloy and spherical cluster phase in Al–0.35Fe–0.2Zr alloy are Al_3_Fe, with diffraction patterns shown in Figure 3b,d, respectively. The results are consistent with the research of Wang, who found that a eutectic Fe-rich phase with a short rod shape is formed during rapid solidification and it is considered as an Al_3_Fe phase [24]. In addition, typical butterfly-like Al_3_Zr precipitation was also found along grain boundary in Al–0.35Fe–0.2Zr alloy.

### 3.2. Effect of Zr Content on As-Cast Tensile Properties

**Figure 4:**
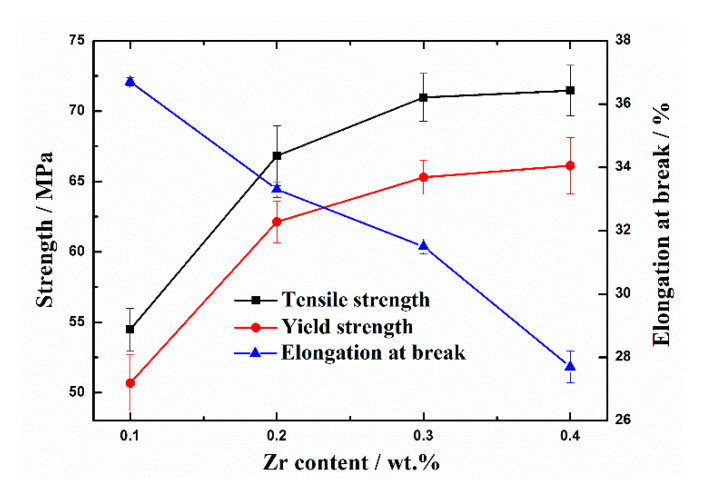
Tensile properties of as-cast Al-Fe-Zr alloy with different Zr contents.

The tensile properties of Al-Fe-Zr alloys with different Zr contents are listed in Figure 4. According to the tensile data error, the mechanical properties of the alloy with different Zr contents are relatively stable, the strength error is ±3MPa, and the elongation is within 1%. It can be found that the tensile strength and yield strength gradually increase and the elongation gradually decreases with increasing Zr content. When the Zr contents vary from 0.1% to 0.2%, the tensile strength gradually increases by 22.8% from 54 to 67 MPa and the yield strength increases gradually by 21.6% from 51 to 62 MPa. However, by further increasing the Zr content, the strength properties increase, but the increment becomes slower. When the Zr content is 0.4%, the tensile strength and yield strength are 71 and 66 MPa, respectively, which are only 6.0% and 6.4% higher than that of Al–0.35Fe–0.2Zr alloy. The elongation decreases with increasing Zr content.

**Figure 5:**
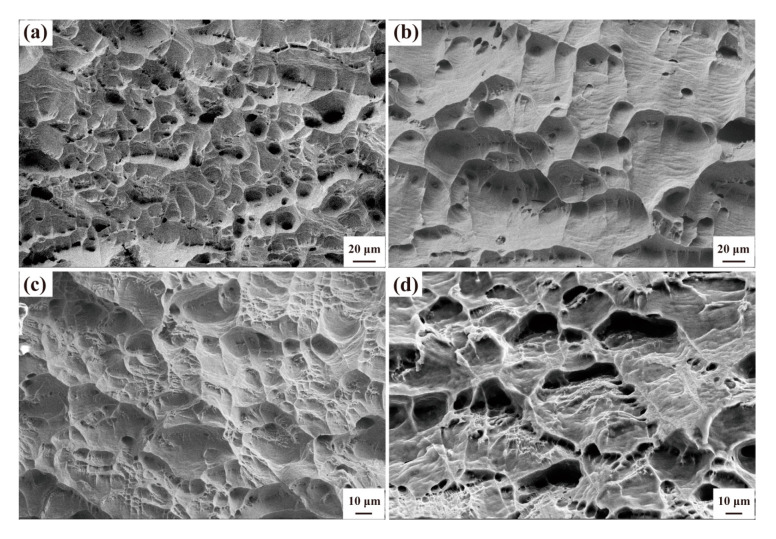
Tensile fracture morphology of as-cast Al-Fe-Zr alloy with different Zr contents: (**a**) Al–0.35Fe–0.1Zr alloy; (**b**) Al–0.35Fe–0.2Zr alloy; (**c**) Al–0.35Fe–0.3Zr alloy; (**d**) Al–0.35Fe–0.4Zr alloy.

As can be seen from the tensile fracture morphology in Figure 5, when the Zr content is 0.1%, the ductile fracture is the main fracture, and the fracture is mainly dimple (Figure 5a). When the Zr content increases to 0.2% and 0.3%, the dimple becomes significantly shallower indicating that the toughness is gradually decreasing. When the Zr content reaches 0.4%, the tearing pattern begins to appear around the dimple, indicating that the toughness is the worst at these alloys. This fracture morphology is consistent with the elongation results obtained in Figure 4, which further indicates that the high Zr content is not conducive to the plasticity improvement.

## 4. Discussion

According to the Al-Zr binary phase diagram, the Zr solutions in the Al matrix present mainly in the form of interstitial solid solutions by means of equilibrium solidification when the content of Zr is less than 0.11%. When the Zr content is above 0.11%, the Al_3_Zr phase is formed during equilibrium solidification [25]. If Zr exists in the form of the solid solution in Al–0.35Fe–0.1Zr alloy, it has the same nucleation effect and can only refine grains by hindering their growth, which limits the refinement effect on the grains. When the Zr content increases to above 0.2%, the Al_3_Zr appears by a peritectic reaction. The morphology of the Al_3_Zr particles is preferentially characteristic of the L12 structure (metastable) [26,27]. The cubic structure of Al_3_Zr is commensurate with a-Al (fcc) and acts as an effective heterogeneous nucleant of a-Al during solidification [28]. Therefore, the grain size of Al–0.35Fe–0.2Zr alloy is significantly refined compared with that of Al–0.35Fe–0.2Zr alloy. With further increasing Zr content, the number of Al_3_Zr particles increases and the grain refinement effect increases, but the change is minimal.

During solidification, Zr mainly concentrates in front of the crystal and hinders Fe atom diffusion effectively. Thus, the growth of primary Al_3_Fe is inhibited and a relatively round short rod shape is obtained [29]. With the consumption of Al atoms, the subcooling zone of the components appears in the interface front. The appearance of the component supercooled zone causes the primary Al_3_Fe phase to be wrapped by post-nucleated Fe-rich phases before it grows [30]. In contrast, the appearance of the component supercooled zone also causes dendrite fusion, separation and proliferation of Al_3_Fe. Therefore, with increasing Zr content, the diffusion resistance of Zr to Fe is enhanced, Al_3_Fe gradually refines and the morphology is gradually formed into clusters composed of shorter rods with smaller sizes [31,32].

The grain size of the as-cast alloys decreases with the increasing of Zr content. The increment of grain refinement on yield strength Δy can be expressed by the Hall–Petch equation [33,34]:

Δy = kd^−1/2^ (1)

where d is the average grain size and k is a constant that is 0.04 MPa·m^1/2^ in Al alloys [35]. The average grain size of the alloys is 76, 35, 27 and 23 μm, respectively. Thus, the increment of grain refinement on yield strength is 4.6, 6.8, 7.7 and 8.3 MPa, respectively. It is found that strength change caused by fine-grain intensification was significant when the Zr content was 0.1 to 0.2%. When the Zr content continues to increase, the strength increment caused by fine-grain intensification changed only marginally.

As a transition element, the concentration of the equilibrium solution of Fe in the Al matrix is 0.0058%. Even under the non-equilibrium solidification of metal mold casting, the solution of Fe in Al can be negligible [1]. The concentration of the equilibrium solution of Zr in Al is 0.11%. Under the same solidification conditions, it can be considered that the Zr content changes from 0.1 to 0.4% and the amount of Zr solution in the alloy is almost unchanged. Zr atoms in the interstitial solid solution can prevent the movement of dislocation and the slipping of grain boundaries. In contrast, Zr atoms can lead to lattice distortion of the Al matrix, which enhances the resistance of dislocation motion and thus increases the strength of the solid solution. The basic rule of solid solution strengthening can be expressed by an equation [36,37]:

(2) $$\nabla \sigma_{sol}\propto \varepsilon_{r}^{3/2}\sqrt{c}$$

(3) $$\varepsilon_{r}=\frac{r-r_{0}}{r_{0}}$$

where *ε_r_* is the lattice misfit, c is the mole fraction of the solute atom, *r*_0_ = 0.143 nm [38], and is the atomic radius of the matrix, and *r* = 0.216 nm [39], and is the atomic radius of the solute. It can be seen that when the amount of solid solution is the same, the strengthening effect of the solid solution in the alloys is basically the same.

Intermetallic compounds possess high hardness and brittleness. They are distributed in the Al matrix, which is equivalent to embedding some irregular hard particles into the soft matrix [40]. Under tension, in order to coordinate deformation, grains in different orientations rotate and this rotation among grains will be hindered by the high hardness phase distributed among grains, resulting in dislocation within grains [25]. As the load continues to increase, the dislocation will start to move. When the dislocation moves to the hard phase interface, it will stop due to the obstruction of the hard phase, thus causing a dislocation plug product at the interface between the hard phase and the matrix. According to the Orowan theory, improvements in yield strength caused by the second relative alloys of Al_3_Zr and Al_3_Fe at the nano or microscale can be calculated by the [41]:

(4) $$\Delta_{or}=M\frac{0.4Gb}{\pi \sqrt{1-\gamma }}\frac{\ln\left(\frac{2r}{b}\right)}{\lambda }$$

where *M* = 3.06 and is the Taylor factor [42], *G* = 25.4 GPa and is the shear modulus [43], *b* = 0.2863 nm and is the magnitude of the Burgers vector [43], *γ* = 0.35 and is the Poisson ratio of pure Al [42], *r* is the mean particle diameter and λ is the mean interparticle distance. Therefore, with the increase in Zr content, the density of Al_3_Zr particles increases and the interparticle distance decreases, resulting in an increase in the increment of yield strength caused by the Al_3_Zr phase.

The plastic deformation of Al alloys is controlled by dislocation slip [44]. However, the hard Al_3_Fe phase can prevent the movement of dislocation and the slipping of grain boundaries, which lead to the phenomenon of stress focus produced at the Al_3_Fe/Al matrix interface and inconsistent deformation [30]. As the deformation progresses, the stress concentration and uncoordinated deformation accumulate continuously. After a certain degree of accumulation, the micro-cracks inside the oxide film gradually expand and link, forming cracks around the hard phase, and the further expansion of the cracks causes the alloy to fracture [30]. As for the short rod shape Al_3_Fe, the stress concentration effect is serious [31,32]. With the increase in Zr content, the volume fraction of Al_3_Fe remains unchanged, but the shape of Al_3_Fe changes from a short rod-like form distributed as a discontinuous network to an agglomerated structure consisting of finer and shorter rod-like shapes. The meshed Al_3_Fe is not as good as the cluster-like Al_3_Fe in deformation coordination. The uncoordinated action between Al_3_Fe and the matrix leads to the occurrence and expansion of cracks, which leads to the fracture of the alloy eventually. Therefore, the tensile strength of Al–0.35Fe–0.1Zr is less than that of Al–0.35Fe–0.4Zr. As the number of strengthening phases increases, the elongation of alloys decreases gradually.

## 5. Conclusions

The microstructure of as-cast Al-Fe-Zr alloys changes as a function of the Zr content. When the Zr content is 0.1%, the alloy is composed of the Al_3_Fe phase and distributing in the Al matrix, and the Zr element exists in the solid-solution state. As the content of Zr continues to increase, nanosized Al_3_Zr particles appear in the alloy.

Zr addition in the Al-0.35Fe alloy refines the microstructure of the as-cast alloy and improves the morphology of the Al_3_Fe phase. As the Zr content increases from 0.1 to 0.4%, the grain size of the Al matrix decreases from 76 to 23 μm. The morphology of the eutectic Al_3_Fe phase changed from a short rod-like shape to an agglomerated structure consisting of finer and shorter rod-like shapes.

The tensile and yield strengths increase with increasing Zr content. When the Zr content increased from 0.1 to 0.4%, the tensile strength gradually increases from 54 to 71 MPa and the yield strength gradually increases from 51 to 66 MPa. The strengthening mechanism of the alloy can be attributed to the combination of fine-grain, solution and second-phase strengthening.

## Figures and Tables

**Table 1:** Actual compositions of the four Al-Fe-Zr alloys (wt.%).

Samples	Fe	Zr	Al
Al–0.35Fe–0.1Zr	0.352	0.122	Bal.
Al–0.35Fe–0.2Zr	0.341	0.247	Bal.
Al–0.35Fe–0.3Zr	0.357	0.298	Bal.
Al–0.35Fe–0.4Zr	0.354	0.380	Bal.
